# Development of the Level of Preventive Action Method by Observation of the Characteristic Value for the Assessment of Occupational Risks on Construction Sites

**DOI:** 10.3390/ijerph18168387

**Published:** 2021-08-08

**Authors:** Antonio José Carpio de los Pinos, María de las Nieves González García, José Antonio Soriano, Benito Yáñez Araque

**Affiliations:** 1 Department of Applied Mechanics and Project Engineering, Toledo School of Industrial and Aerospace Engineering, Campus of International Excellence in Energy and Environment, University of Castilla-La Mancha, Real Fábrica de Armas, Edif. Sabatini. Av. Carlos III, s/n, 45071 Toledo, Spain; joseantonio.soriano@uclm.es; 2 Escuela Técnica Superior de Edificación, Universidad Politécnica de Madrid, Avenida Juan de Herrera, 6, 28040 Madrid, Spain; mariadelasnieves.gonzalez@upm.es; 3 Applied Intelligent Systems Research Group, Department of Physical Activity and Sports Sciences, Campus of International Excellence in Energy and Environment, University of Castilla-La Mancha, 45071 Toledo, Spain; Benito.Yanez@uclm.es

**Keywords:** health and safety, risks assessment, construction, characteristic value, preventive environment

## Abstract

The special circumstances of the high accident rate in the construction industry compared to other sectors are significant and represent a major concern for many countries. Construction work involves a large number of risks that cause or may cause accidents with serious consequences for the worker’s health, even death. The Level of Preventive Action is a novel methodology of occupational risk assessment adapted to building works. It is based on the development of the mathematical formulation of William T. Fine’s method. Its implementation covers four of the techniques for combating risk: Safety at Work, Industrial Hygiene, Ergonomics and Psychosociology. It evaluates, quantitatively, the amount of preventive action required based on the characteristic complexity of the work units, their location and their interdependence. The method protocol defines a new observation parameter called Characteristic Value which is inherent to the real situation of the construction process. The aim of this study is to develop the characterisation of the Characteristic Value in the Level of Preventive Action method. It also justifies the procedure to obtain this Characteristic Value and how its implementation and result should be interpreted. Finally, the methodology is applied on a real case.

## 1. Introduction

The construction sector has the highest number of occupational accidents, which highlights the gap between the application of the law and the workers’ own liability in the event of an accident or incident [1,2,3,4]. Construction work involves a large number of risks that cause or may cause very serious and fatal accidents [5,6,7]. The construction systems of excavation, earthmoving, construction, assembly and disassembly of prefabricated elements, fitting out installations, transformation, rehabilitation, repair, dismantling, demolition, maintenance, conservation, painting, cleaning or sanitation work, site meetings, weather conditions and rushed delivery times are the general tasks with the highest incidence of accidents in construction [8]. The reduction of occupational accidents in the social sphere is a priority objective. Consequently, accident prevention, risk assessment and risk management are decisive issues in this sector [9,10]. Occupational risk assessments often analyse specific risks, but the occupational accident rate must be analysed from a global approach [11,12].

Training in the observation of workers at their workstations is a fundamental preventive activity to identify unsafe or deficient acts [13,14]. Given the difficulty in the economic and social sphere, accidents at work represent permanent economic losses for companies, administrations, workers and society in general [15]. It is essential to identify and assess the seriousness of the risk in order to anticipate the order of action in terms of prevention. For this reason, the probability of the damage occurring, and the seriousness of its consequences must be analysed together [16].

The occupational risk assessment procedure in construction work is being developed and adapted to the characteristics of the building process [17], from the initial approaches to prevention in business action and project conception [14], through the study of prevention in the project phase and during the construction phase of a building [18]. The study of the safety climate in social environments and human behaviour in terms of prevention must also be taken into account [19]; with the essential study and analysis of psychosocial risks throughout the hierarchical structure of companies [20] and, finally, during the use and maintenance phase of the building with the new owners [21]. However, these assessment procedures still do not cover all risk control techniques because of the complexity involved in their direct application on site [12,22]. It is essential to identify the preventive parameters for a construction site and to encompass the different risk control disciplines [12,22], which are active during the construction procedures and the development of a building:

- Safety at Work, which aims to prevent accidents at work in which there is direct contact between the material agent (equipment, substance, product or energy) and the worker with traumatic consequences (burns, wounds, contusions, fractures, amputations, etc.). These are risk factors derived from the place and surface of work, machines and work equipment, electrical risks, fire risk, handling and transport [23].
- Industrial Hygiene, which assesses the hygienic risks in the workplace. It is the key tool to address the elimination, reduction and control of exposure to chemical, biological and physical agents through preventive planning [24].
- Ergonomics, aimed at understanding the person’s capacity for adaptation, which is identified within a narrow comfort zone, and which is what this technique aims to preserve. It studies the adaptation conditions of a workplace, a machine, a vehicle, etc., to the physical and psychological characteristics of the worker [1,25].
- Psychosociology, as psychosocial risks are part of the so-called emerging risks, as important or more important than any of the better known or classic risks (health and safety) and how they are caused by poor working conditions [10,26]. Psychosocial risks are consequences of deficiencies in the design, organization and management of work. They are also due to the low social context of work, and can lead to negative psychological, physical and social outcomes, such as job stress, burnout or depression [27].

However, a clear evolution of the evaluative aspects of the new risk assessment methodologies can be observed, which have been gradually encompassing and incorporating different areas of research [12,18,19]. Current risk assessment methodologies focus on the evaluation of broader aspects due to the fact that the cause or reasons that generate an accident are not usually particular, but rather depend on various circumstances in the work environment (safety aspects in the work environment) such as work, occupational disease, adaptation to work or social relationships. Many investigations determine the need to evaluate globally and covering all possible study disciplines [21,22,23].

Based on these developments, current methods of occupational risk assessment are increasingly adapted to the particular characteristics of construction sites [28]. The current interest is in taking global data on all the risks specific to construction sites [29] and the uncertainty caused by the action of the individual [30]. Such is the case, as in the method proposed by Forteza et al., which obtains information on the construction environment, its structure, its development over time, the workers and the type of construction site; identifying and quantifying the risks, barriers and means based on dichotomous criteria (presence/absence, right/wrong, 1/0, yes/no) [31]. The evaluation method proposed by Pinto with the development of a multidimensional Event Tree based on linguistic variables on four observation approaches: safety climate, severity, possibility and means of safety; quantifies the characteristic risks of construction sites with a range of values from 0 to 1 [32]. A very different approach is proposed by Simanaviciene et al., considering the uncertainty associated with decision-making in risk prevention aspects, following a flow diagram and establishing selection criteria within a range of values between 0 and 1 [33]. Continuing with social aspects, Salanova et al. develop organisational and individual work stress prevention strategies in order to optimise the health and well-being of the company organisation, warning about workers’ overconfidence as a risk parameter and measuring within a spectrum between positive or negative situation [34]. Due to the scarce documentation on occupational risk prevention in construction projects and its enormous incidence in occupational accidents [18,35], Reyes et al. analyses the sustainability of construction processes. To this end, it offers the possibility of minimising accident rates and reducing project costs, covering the four phases of a building’s life cycle: design, construction, useful life and reintegration; covering a range of values from 0 to 100 [36]. A broader assessment is proposed by Oliveira justifying that the consequences of risk are based on human error, machine failure, social environments and individual health. He justifies this on the basis of four types of event trees that determine the risk situation: cause tree, decision tree, effect tree and failure tree; with a range of values between 0 and 1 [37]. Finally, Claudino proposes a risk assessment methodology based on the conformity and adequacy of compliance with prevention regulations, identifying the hazard and the workers associated with this circumstance, with a range of values from 0 to 1 [11].

Despite the existence of various risk assessment systems, no tools have been found that make it possible to obtain an overall assessment of a construction site as a whole [24,25]. In this sense, the prevention of occupational risks in construction must be analysed from different points of view. Firstly, in order to combat occupational accidents, it is essential to integrate risk prevention into all phases of business activity [38]. Many studies have shown that one of the key factors in the prevention of risks in a building site is a correct study of the health and safety conditions in the design phase [39]. In turn, the management of risk prevention during the execution of a building is fundamental [8], and it is essential to focus on health and safety outside of bureaucratic and economic roles, with greater involvement of all the agents that make up the construction [38]. Regarding the study of the safety climate in companies, an alternative approach to risk prevention is offered [40], incorporating the uncertainty generated by human behaviour [41]. This generates a mismatch between the worker and the working conditions [11], alerting construction workers to the most frequent dangerous procedures and praising safe work situations [42]. Finally, the stage of occupational risk prevention during the use and maintenance phase of the building should be extended, as within the scope of the building’s life cycle there are factors at play that require preventive procedures [43]. Based on the diversity of points of view, risk assessment is necessary from the joint aspects of Occupational Safety, Industrial Hygiene, Ergonomics and Psychosociology [44], and the necessary parameters must be established for a correct assessment of occupational risks, in accordance with the particular characteristics of construction sites [12,45,46].

This new methodology assesses risk from different aspects: occupational safety, industrial hygiene, ergonomics and psychosociology. In addition, it analyzes how risk is understood differently with respect to the observation environment: documentary, constructive, and social. This study analyses the particular parameters of the occupational risk assessment methodology adapted to construction sites, known as the Level of Preventive Action. Within this analysis, the need to establish a new quantifiable observation criterion that adapts to the construction reality is raised. This new criterion is called Characteristic Value and is characterised by giving a greater approximation to the quantification values of the associated risks for each construction system. Finally, the Level of Preventive Action determines, quantitatively, the amount of preventive action that is required to reduce the risk situation in a global or particular way in all or in each of the evaluation disciplines: Work Safety, Industrial Hygiene, Ergonomics and Psychosociology; and in all or in each of the risk assessment environments: documentary, constructive and social.

Methodologically, this work proposes the definition of the Level of Preventive Action (*Lpac*) method, the preventive observation environments, as well as their corresponding parameters [12,46]. Then, the new observation parameter for risk quantification, called Characteristic Value (*Cv*), is defined, and justification is given for the range of values it takes. Based on the *Lpac* protocol [12,45], the implementation on a real construction site and the interpretation of the results with respect to Characteristic Value (*Cv*) is carried out.

## 2. Level of Preventive Action Foundations

Previously, the theoretical foundations of this new methodology are defined, which is based on the mathematical formulation proposed by William T. Fine [47]. New concepts of preventive observation called preventive environments and the definition of new parameters of the level of preventive action are proposed. The direct and inverse relationship between the different parameters is determined. A new risk assessment formula called level of preventive action is proposed, which determines the amount of preventive action that is required to ensure that the risk situation is optimal.

### 2.1. Theoretical–Mathematical Foundations

The Level of Preventive Action (*Lpac*) is an occupational risk assessment methodology adapted to building works [12,45,46], adjusted to the “special” complexity of these works. Its implementation is based on a mathematical formulation developed from William T. Fine’s method [46,47]. This method was defined as “Mathematical Evaluation for Controlling Hazards” and was published in 1971 by the North American Naval Ordnance Laboratory. This method determines a formula that relates the control factors, achieving a numerical evaluation of the importance of the corrective measure of the hazard. That allows to establish the priorities of correction of the preventive action. On the other hand, the justified cost parameter is determined by the estimated cost and the effectiveness of the corrective action towards the risk. It is worth highlighting this method, which was developed in the naval instruments sector, and in the methodology itself, William T. Fine, makes absolute mention that it could be universalized by making the adaptations and corrections that were considered pertinent [47].

In general, the new methodology of the Level of Preventive Action establishes criteria similar to those defined by the William T. Fine method. It incorporates parameters based on the stages of the construction process according to the risks associated with these stages (Table 1). The table establishes the criteria vertically. The construction stages are established in five sections: initial design, project drafting, contractor contract, project implementation and use and maintenance. Next, the corresponding risks associated with the construction processes of each stage are analyzed. In the first, traditional risks (probability and consequences) are analyzed. In the second, the risks associated with the physical conditions of the materials, the training of the workers, and the geometric conditions of the building are analyzed. In the third, the risk factors that are generated in the process of contracting construction work with the contractor, which depend on construction planning, construction resources and preventive systems. In the fourth, during the implementation of the project, psychosocial risks are analyzed with emotional states and the participatory interest of all workers in risk prevention. In the fifth stage, the associated risks are defined by the concepts of use and maintenance, which include documentary risks, risks of use, maintenance risks, risks of neighborhood relations, economic risks and legal risks. These parameters are not inherent to the construction process, so a more detailed study should be referred to outside the scope of the execution of the work [46].

The *Lpac* parameters observe the reality of a building site in each of the preventive environments of the building process: initial, documentary, constructive and social [12,46]; comprising four of the techniques for combating risk: Safety at Work, Industrial Hygiene, Ergonomics and Psychosociology. The new risk assessment method establishes the amount of prevention level that is deviating from the initial approach, in the Occupational Health and Safety Plan, determining the amount of preventive action that needs to be incorporated into the development of the work to improve the design conditions, constructive conditions and social relations, in the initial environment. This observation determines, quantitatively, the risk levels that correspond to the complexity of the work units, their location on the site and their interdependence [48], in the documentary environment [38]. It also determines, quantitatively, the risk levels according to the characteristics of the construction systems and preventive systems [15], in the construction environment [44]. Finally, it determines, quantitatively, the levels of risk based on the perception of the environment and the mood of the workers [10], in the social environment [49].

Thus, in this methodology, the parameters that define the *Lpac* cover the first four stages of construction:

- In the initial or absolute environment (*Abe*), the parameters are probability (*P*) and consequences (*C*) as basic risk parameters.
- In the documentary environment (*De*), physical parameters are described with the relative risk (*Rr*) and geometrical parameters of the building with the border risk (*Br*).
- In the constructive environment (*Ce*), the degree of exposure to risk of the worker (*E*) and the economic capacity in prevention provided by the company (*Ec*) are measured.
- In the social environment (*Se*), the parameters of the participative interest in prevention (*Pi*) and the level of worker satisfaction (*Ls*) are measured.

The mathematical expression that defines the *Lpac* based on the assessment parameters of the preventive environments, is as follows:

*Lpac = (Abe) · (De) · (Ce) · (Se),* (1)

The parameters corresponding to the detailed assessment of each of the environments and their interpretation according to the degree of correction (by direct or indirect relationship) are as follows:

*Lpac = (P · C) · (Rr · Br) · (E · (1/Ec)) · ((1/Pi) · (1/Ls)),* (2)

The mathematical expression of *Lpac* is as follows:

*Lpac = (P · C) · ((Rr · Br · E)/(Ec · Pi · Ls)),* (3)

From the mathematical expression that defines the *Lpac*, it is interpreted that a third corrective parameter called Evaluation of Preventive Action is applied to the probability and consequences parameters:

*Lpac = (P · C) · (Apac),* (4)

*Apac = ((Rr · Br · E)/(Ec · Pi · Ls)),* (5)

Everything exposed in this section can be consulted in the doctoral thesis of the new methodology [12] and in the publication of the article on the theoretical–mathematical foundations of the method [46].

### 2.2. Level of Preventive Action Protocol

An outline of the protocol of the *Lpac* methodology is shown (Table 2). The action protocol at *Lpac* is based on specialised technical observation, specialized technical analysis, and data collection with regard to techniques for combating work safety, industrial hygiene and ergonomic risks, and a psychosocial survey on site [12,45].

It comprises five fundamental phases that analyse the actual situation observed during data collection. The first phase of the protocol defines a *Cv* inherent to the observed site situation, in the absolute, documentary, constructive and social environments; and is applied to each of the parameters of the Level of Preventive Action formula. The *Cv* corresponds to those defined in the project in the absolute environment. The *Cv* in the documentary and constructive environments corresponds to a specialized technical evaluation. The *Cv* in the social environment corresponds to a survey in the workplace. The second phase assesses the incidence on the assessed risk of the documentary environment, constructive environment and the social environment. It is analyzed how the *Cv* of the *Apac* parameters varies with respect to the evaluation of a risk. The assessor must decide which risks assessing: Work Safety risks, Industrial Hygiene risks, Ergonomic risks and/or Psychosociology risks. The third phase indicates the basis of prevention control with the obtained value of the *Lpac* in relation to the absolute risk (*Abr*), as a deviation from the initial preventive action. For a better interpretation of the value obtained, the unit of *Lpac* is in percentage. Following the same curve that identifies the characteristic values, and in a proportional way, the different levels of preventive action control are identified. The fourth phase indicates the recommendation actions in terms of risk prevention. The preventive action recommendations can be individual or group, and cover all levels of risk assessment, all levels of environmental assessment, and all levels of worker assessment. Furthermore, in the fifth phase, the improvement of preventive action during the construction process is checked [12,45]. This check can be individually or collectively.

Everything stated in this section can be consulted in the doctoral thesis of the new methodology [12] and in the publication of the article on the protocol for applying the method [45].

### 2.3. The Characteristic Value

The *Cv* is associated with the characteristics of the work unit under execution [24] on which it is to be assessed, on each of the parameters of the *Lpac* and based on technical observation criteria such as the complexity of the work unit [2], the location of the work unit, degree of exposure to risk, organisational procedure in risk prevention, participation in risk prevention of workers and congruence of risk perception between the worker and the assessor [50]. The *Cv* positions, with the integer values 1, 3, 5, 9, 15 and 25, the degree of risk. This *Cv* will be the base or reference value of the risk associated with the real conditions of the site. The impact of this *Cv* on each of the risks to be assessed is then evaluated, the result of which may increase or decrease [12,45]. The value corrected according to its impact on the risk assessed provides a value that is transferred to the corresponding parameter of the *Lpac* formula. The results determine, with quantitative criteria, the percentage or amount of preventive action required. Therefore, the *Lpac* method is more flexible in its applicability and more sensitive to detect risks in all situations in the construction process.

**Figure 1:**
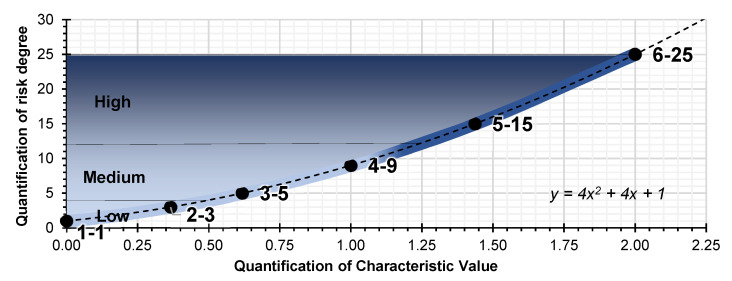
Characteristic values graphic: relative risk, border risk and exposure risk.

The observation criterion for data collection, for each of the parameters, is divided into three contexts with respect to the degree of risk observed: low risk, medium risk and high risk (Figure 1). This criterion is of great help in interpreting the observation and in determining the *Cv* correctly and efficiently. Each observed risk degree context is assigned a dual qualitative value (easy, difficult; a little, a lot; less, more; etc.) and easily quantifiable. Each criterion analysed on the unit of work assessed will have a degree of risk, a priori, which is identified as low and high; and its corresponding *Cv* from lowest to highest. 

In risk assessment, the difficulty is in adding a quantification value to the qualitative value. Normally, it is usually indicated that there is a lot or little prevention, that more or fewer preventive systems are needed, etc. Much information is collected during the site inspection. Therefore, those workers can answer in a simple way, and it is better to propose simple (dual) conditions that are easy to quantify by the evaluator. The values on which the worker has to decide are 1-2-3-4-5-6, divided into low (1–2), medium (3–4) and high (5–6). For example, the worker is asked about the conditions of collective protections in the work. To help him in the answer, three alternatives are proposed: low, medium, and high. If the worker decides that it is medium, he is asked again if it is medium-low (3) or medium-high (4). The values given by the worker, 1-2-3-4-5-6, correspond to the characteristic values 1-3-5-9-15-25. The former is more intuitive and easier to interpret by most workers. This makes the worker aware of the preventive environment of the work. It is also avoided that the result is conservative and always stays in the middle.

**Figure 2:**
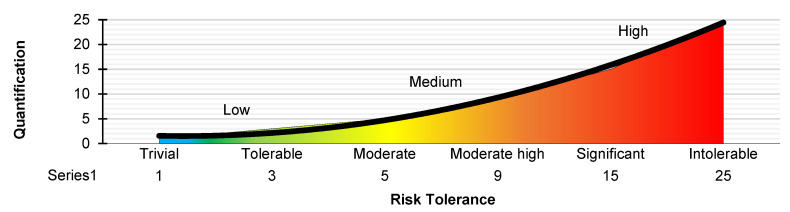
Characteristic values graphic: relative risk, border risk and exposure risk.

**Figure 3:**
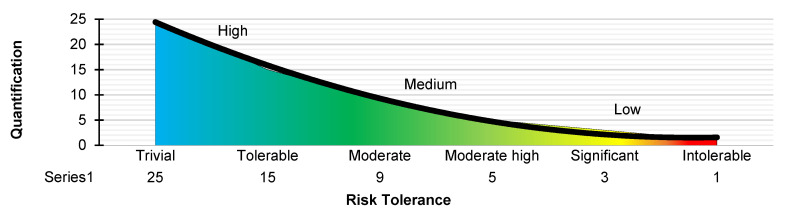
Characteristic values graphic: economic capacity, participative interest and satisfaction level.

Generally, the risk assessment in the Occupational Health and Safety Plan drawn up by the construction company uses qualitative parameters of the general method, published by the INSST, which estimates the risk tolerance [51] (INSST means in its Spanish description: “Instituto Nacional de Seguridad y Salud en el Trabajo”, in English: “National Institute of Health and Safety at Work”). The *Lpac* method applies a matrix of results that quantifies the linguistic evaluation criteria of the general method. The three numerator parameters in the *Lpac* parameter have a direct proportional relationship in line with the complexity of the site, the location of the workers and their degree of exposure to risk. The higher the *Cv*, the greater the a priori risk involved. The reading and interpretation is the same for probability and consequences (Table 3). The scale of values of the a priori risk estimate for the *Cv* is 1, 3, 5, 9, 15 and 25, from trivial to intolerable (Figure 2). The three denominator parameters in the *Apac* have an inverse proportional relationship in line with the correction levels provided by economic capacity, participation in prevention and the *Ls* of the workers. The higher the *Cv*, the higher the degree of a priori correction there is. The reading and interpretation are the reverse for probability and consequences (Table 4). The scale of values of the a priori risk estimation for the *Cv* is 25, 15, 9, 5, 3 and 1, from trivial to intolerable (Figure 3). 

The quantification values in Table 3 and Table 4 (probability and consequences) are based on the quantification of the risk tolerance matrix: trivial risk, tolerable risk, moderate risk, significant risk, and intolerable risk. The quantization values are 1, 3, and 5; whose mathematical function is *f (x)* = 2*x* + 1, for the range of integer values 0, 1 and 2. In turn, the *Cvs* are the result of the quantized probability and consequences matrix. The quantization values are 1, 3, 5, 9, 15 and 25, whose mathematical function is *f (x) =* 4*x*^2^ + 4*x* + 1, for the range of values [0,2].

All the statements and the quantification values and their corresponding justification, can be consulted in the doctoral thesis of the new methodology [12] and in the publication of the article on the protocol for applying the method [45].

The risk assessment factor contains parameters that increase or decrease the characteristic value. In turn, the assessment criterion is ascending from lowest to highest in the parameters of absolute environment, documentary environment, built environment and social environment. Therefore, the parameters with direct proportionality will increase their result for high *Cvs*, due to greater complexity, greater borderline risk, and greater risk exposure. Furthermore, the parameters with inverse proportionality, for high *Cvs*, will correct the situation with greater organisational procedure, greater *Pi*, and greater Ls. In the first phase of the methodology, the observable reality is quantified by means of the Cv. The range of values is 1, 3, 5, 9, 15 and 25, and is assigned to each parameter of the formula.

## 3. Interpretation on a Case Study

**Figure 4:**
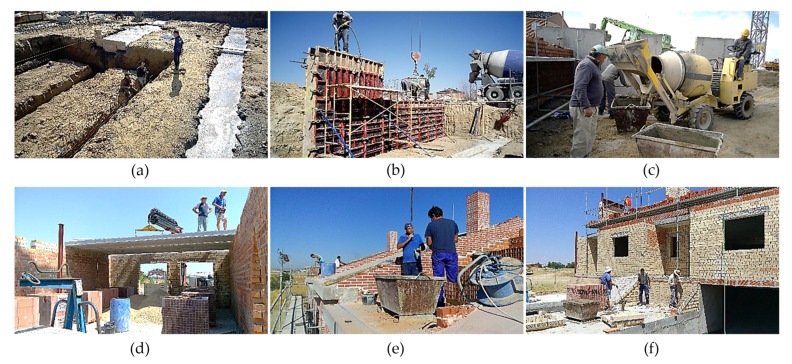
Different moments in the construction process of the building: (**a**) Foundation; (**b**) Reinforced Concrete Walls; (**c**) Urbanisation; (**d**) Precast Slabs; (**e**) Roofs; (**f**) Facades.

**Figure A1:**
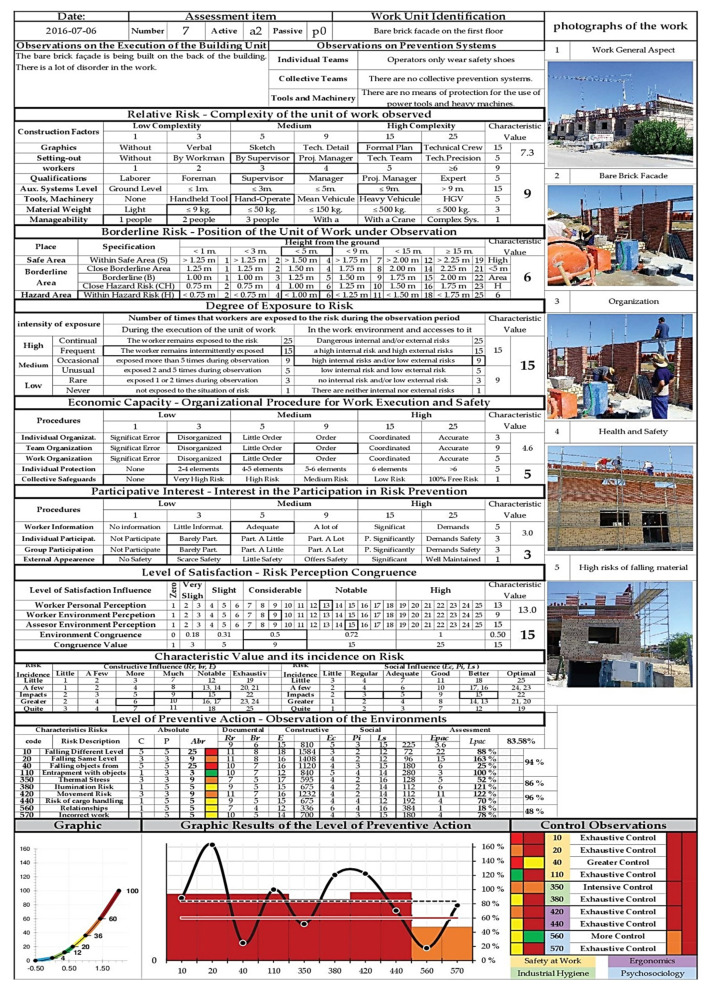
Help sheet for data collection and calculation of the characteristic value, its incidence on the risk, level of preventive action, and preventive action controls.

**Figure A2:**
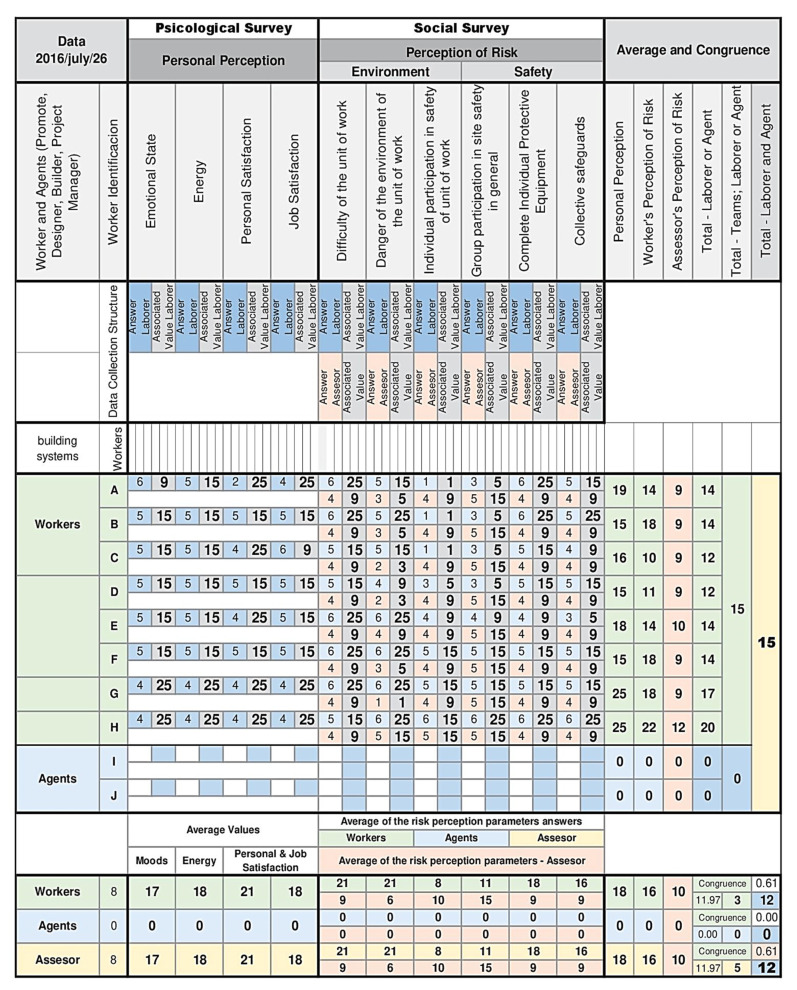
Help sheet for the survey for the calculation of the satisfaction level (Ls) parameter and the interpretation of results for the participatory interest (Ip) parameter.

Data have been taken from a real construction process located in the province of Madrid (Spain). It is a building for six dwellings on three levels (a semi-basement floor and two floors for the dwellings). The total built area of the building is 1528.26 m^2^. From 17 June 2016 to 27 April 2017, data collection was carried out on a weekly basis for a total of 34 working weeks. In total, 74 risk evaluations of the construction systems and 34 surveys of the workers who were working in each inspection have been carried out. Appendix A shows two images (Figure A1 and Figure A2) that were used to collect data from the evaluations of the construction systems and collect data from the surveys. In both cases, correspond to 26 July 2016. Some photographs of the development of the work are shown (Figure 4). The occupational health and safety context observed during the construction process was very delicate, with obvious high-risk situations. Technical data were collected by direct observation and psychosocial data were collected by means of a site survey. The questionnaire asked workers and site agents (promoter, planner, builder, site manager and health and safety coordinator) about their perception of risk from different points of view on the construction systems: individual and group perception of risk, perception of the controlled risk of the site unit and the environment, and perception of individual and collective protections.

**Figure 5:**
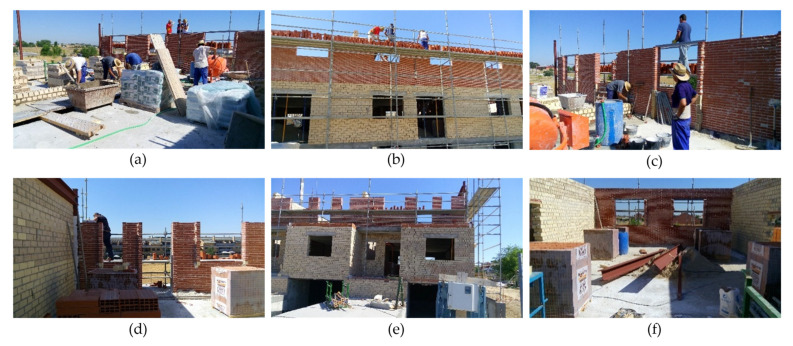
Different moments of the work unit in the case study: construction of an exposed brick façade: (**a**) Site Organisation; (**b**) Work on Scaffolding; (**c**) Personal Protection; (**d**–**f**) Fall Risks.

Section 3.3, Section 3.4, Section 3.5, Section 3.6, Section 3.7 and Section 3.8 analyse the *Cv* corresponding to each of the *Lpac* parameters applied, specifically, to a construction unit of a brick façade on the first floor (Figure 5). In the first phase of the assessment, an a priori degree of risk is quantified for each of the *Apac* parameters.

### 3.1. Probability Parameters and Consequences

There is a large literature on the definition of risk (*R*) and the parameters of which it is composed. Most accept the direct relationship between the probability (*P*) of an accident occurring and the consequences (*C*) of the accident as the product of the probability and the consequences (*R = P · C*) [49,52,53,54,55,56]. However, there are research that includes another corrective parameter for the exposure (*E*) to the risk, with the expression *R = P · C · E* [12,57]. Finally, William T. Fine proposes incorporating another corrective parameter based on the degree of risk correction (*G*), resulting in the expression *R = P · C · (E/G)*, which is closer to the real circumstances [46,55]. However, these parameters have a qualitative and quantitative definition that is very difficult to interpret and apply on construction sites [22,58].

Probability and consequences are parameters that define the risk of a situation. They are determined in the risk assessment of the Occupational Health and Safety Plan drawn up by the contractor. To select the risks to be evaluated, the risk classification published by the INSST (Spanish National Institute for Occupational Safety and Health) has been used [51]. For this research, 10 risks have been designated following the proportionality of risks defined by the INSST: four Safety at Work risks (codes: 010-020-040-110); two Industrial Hygiene risks (codes: 350-380); two Ergonomic risks (codes: 420-440) and two Psychosociology risks (codes: 560-570). The column “Health and Safety Plan. Assessment Company” identifies the risk assessment determined by the Health and Safety Plan defined for the construction of the building. These qualitative values are called “risk tolerance” and are based on the general risk assessment method defined by the INSST. The evaluator matches the qualitative values of “risk tolerance” with their corresponding *Cvs* of the new evaluation method (*Lpac*). They are values that serve as a basis for establishing prevention systems during the execution of a building. Their value will serve as a comparative basis for the rest of the parameters, so it acquires a conceptual value of absolute risk: *P · C = Abr*. It is the evaluator’s mission to interpret the risk assessment of the Health and Safety Plan. The probability and consequences parameters will be matched with a *Cv* (Table 5). 

### 3.2. Parameters of the Preventive Action Evaluation

Next, we proceed to identify the observational criterion for each of the *Apac* parameters (Table 6). All the values shown in the table correspond to the data collection sheet of 6 July 2016 (Appendix A, Figure A1). To do so, the criterion of the *Lpac* methodology protocol is followed [12,44]. The unit of work on which different observation elements are applied is observed. Each element is evaluated with the values 1, 3, 5, 9, 15 and 25 according to its greater or lesser degree of presence on the site and with respect to the specialised observation of the evaluator. 

In this way, parameters of a physical and geometric nature are evaluated in the documentary environment (in the relative risk: graphism, setting-out, workers, qualification, work plan, machines, material weight, manageable; and in the border risk: the work height and the distance to the border). In the construction environment, the intensity and frequency of exposure to risk, site organisation and preventive protection are evaluated (in the degree exposure: the intensity and the number of repetitions; and in the economic capacity: the individual and group organisation, work organisation, and equipment personal and collective protection). Further, in the social environment, information, participation, state of mind and perception of the preventive environment are evaluated (in the interest participatory: worker prevention information, individual and group participation in prevention, and external appearance of the construction work; and in level of satisfaction: emotional state with the personal perception, safety perception and the environment perception). The average value is obtained and rounded according to the criterion described in the protocol. The following subsections refer to the *Cv* obtained for each of the *Apac* parameters.

### 3.3. The Characteristic Value of the Relative Risk Parameter (Rr)

It is necessary to incorporate a parameter that establishes an evaluation criterion based on the complexity of the work unit being evaluated, independently of the prevention conditions and economic conditions. The same work unit may offer different conditions in relation to the interdependencies of the organisation and processes of the construction systems of work execution. The *Rr* is the parameter of the *Apac* term, which interprets the construction complexity of the construction unit, and its degree of correction increases the value of the absolute risk. These parameters help to interpret the conditions of the work unit and how they can become conditioning factors of accident risks during the execution work. Based on the observation, a quantified *Cv* is determined which, a priori, marks the amount of complexity of the work unit. Using the *Lpac* Protocol [12,45], the *Cv* of the *Rr* to be evaluated on the basis of the work unit of execution of a brick façade on the first floor (Figure 5). In the case study (Table 6), the result of nine indicates is that the pre-risk estimate or *Cv* is moderately high. The probability of the accident occurring is medium with harmful consequences. The *Rr* assessment is characterised by the analysis of graphical information, layouts, number of workers, qualification, auxiliary work systems, use of tools and machines, weight of material and handling.

### 3.4. The Characteristic Value of the Border Risk Parameter (Br)

Research on the most common risks in the construction sector establishes falls to the same or different levels due to inappropriate postures as one of the main risks [59]. The research suggests changing the meaning of risk and its control with regard to falls from height. It calls for the need to establish criteria that determine the location of the risk of falls [40]. Such criteria would be conditioned by height or vertical risk and by distance or horizontal risk. The *Br* is the preventive parameter that interprets the location of the work unit and its incidence by the surroundings, and which increases the absolute risk value. In this parameter, two points of view are analysed, based on the possibility of a free fall: the actual height from the working plane to the ground; and the location of the operator in relation to the dangerous situation. This concept depends on the theoretical distance from the worker(s) to the hazardous situation. The *Cv* identifies the intermediate zone between the safe zone and the unsafe zone, which is called the boundary zone.

Using the *Lpac* Protocol [12,45], the observation parameters and their quantification are determined. The degree of risk due to height and location will always exist; therefore, the *Cv* of the frontier risk establishes an a priori criterion of the risk that exists depending on the location of the construction site (Figure 5). This value does not analyse the amount of individual or collective prevention elements that the site has. Any construction element that is more than one metre high or one metre deep will have a degree of risk. In the case study (Table 6), the result of six indicates that the estimate of the previous risk or *Cv* is moderately low. The probability of the accident occurring is low with extremely damaging consequences or with a high probability of the accident occurring with slightly damaging consequences. The *Br* assessment is characterised by the location of the worker at different levels of height or depth from the ground and by the separation of the worker from the slab edge or excavation edge.

### 3.5. The Characteristic Value of the Degree of Exposure (E)

The *E* of the worker to certain situations at the workplace means that the probability and possible consequences of an accident vary [60]. It is also accompanied by discrepancies in workers’ concepts and interpretations of occupational accidents when planning prevention programs or improving accident information records [61], with exposure being a determining element in the occurrence of accidents. The *E* is a parameter that evaluates the amount of time that is spent to complete the work unit, and that therefore, the worker is exposed to the risk several times during the development of the work unit. It is very difficult to determine exactly how many times a worker is exposed to a risk situation. However, during the observation or inspection time, it is possible to observe the movement of the workers and to determine this degree of exposure with relative precision. This assessment can be carried out on a single worker, on a team of workers or on the whole site.

Using the *Lpac* Protocol [12,45], the observation parameters and their quantification are determined. The degree of risk due to the intensity of exposure (continuous, frequent, occasional, unusual, rare and never) and the number of times this exposure is repeated during the observation time (in the site unit, around the site and at the accesses to the site unit), establishes an a priori criterion of the risk that exists depending on the location of the site (Figure 5). In the case study (Table 6), the result of 15 indicates that the estimate of the prior risk or *Cv* is remarkable. The probability of the accident occurring is high with harmful consequences or with a medium probability of the accident occurring and with extremely harmful consequences.

### 3.6. The Characteristic Value of Economic Capacity (Ec)

It is in the construction sector that occupational accident rates are higher than in other sectors. Human and financial factors have the greatest impact on the costs associated with these accidents [62]. It is, therefore, essential to integrate safety on the construction site, use safety systems and respect the established economic plan [55]. The *Ec* is a parameter that evaluates the organisational procedure of the construction site execution and the observation of the amount of economic means used in the construction prevention systems. It is a value that decreases (corrects) the absolute risk parameter. The evaluation analyses the individual, group, and site organisation as well as the number of individual and collective protection systems.

Using the *Lpac* Protocol [12,45], the assessment of the parameter establishes an a priori criterion of the risk that exists as a function of the organisation and the number of protection systems. In the case study (Table 6), the result of five indicates that the prior risk estimate or *Cv* for the degree of correction is moderately low. The probability of an accident occurring is high with little harmful consequences or with a low probability of an accident occurring and with extremely harmful consequences.

### 3.7. The Characteristic Value of the Participating Interest (Pi)

It is widely accepted that unsafe behaviour is intrinsically linked to accidents at work. Construction workers’ attitudes towards safety are influenced by their perceptions of risk, management, safe roles and procedures [44]. Correct or incorrect organisation of preventive systems can influence perceptions of the safety climate, and these perceptions influence safe action through their effects on knowledge and motivation [63]. It is, therefore, essential to establish the basis for participation in prevention systems in employers and workers [63], focusing on health and safety outside bureaucratic and economic roles, with the need for greater involvement of all the agents that make up construction [15].

The *Pi* is a parameter that evaluates the participation in prevention of the different agents involved in a construction site by obtaining a perception of health and safety. This parameter is based on observation and conversations with workers. The information that the worker has on prevention, training courses, knowledge of occupational health and safety regulations and whether he/she requests or demands that he/she be provided with individual and collective means of protection is analysed. Next, the involvement and participation of the worker in the prevention procedures for the site is analysed in his own work unit, individually, and in the rest of the work units at group level. Another aspect to consider is the participation of the company and the workers with respect to the outside of the site. The aim is to ensure that the preventive systems cover the immediate surroundings of the site, for the benefit of the residents of the area. 

Using the *Lpac* protocol [12,45], the assessment of the parameter establishes an a priori criterion of the risk that exists depending on the participation of workers in prevention. In the case study (Table 6), the result of three indicates that the estimate of the prior risk or *Cv* for the degree of correction is remarkable. The probability of an accident occurring is high with harmful consequences, or the probability of an accident occurring is medium with extremely harmful consequences.

### 3.8. The Characteristic Value of the Level of Satisfaction (Ls)

Recent research has analysed psychosocial risks as very important elements to be taken into consideration in prevention systems during the construction phase of a building. The different work environments, the worker’s ability to perform the task and his or her motivation must be identified as factors that are directly involved in accidents at work [64]. Similarly, it is essential to incorporate the evaluation of psychosocial risks in the building process to measure the well-being of workers, the overconfidence of experienced workers, the inexperience of young people, routine and overload [50], to establish prevention strategies that alert workers to unsafe behaviours [42] and to ensure that prevention behaviours are appropriate on the basis of higher levels of participation [65]. More and more studies are analysing the relationship between happiness and productivity in the evaluation of *Ls* [66]. The *Ls* is a parameter that considers general aspects of human behaviour, mood and attitude that influence, or can influence, in a decisive way the generation of risks. This parameter is carried out by means of an on-site survey of all the workers who are participating in the work. The questions cover the criteria of personal perception, perception of safety and perception of the environment. This parameter assesses stress and mood in general. Stress is a very common phenomenon in today’s society. A distinction is made between eustress or positive stress (optimal level of activation to perform activities), which has a protective function for the organism, and distress or negative stress (excessive or inadequate level of activation of the organism), which causes dysfunctions in the person. Mood states cause human behaviour to alternate between eustress and distress, due to tension and fatigue; affecting health, nutrition, sleep time, physical exercise and daily development [67].

Using the *Lpac* protocol [12,45], the assessment of the parameter establishes an a priori criterion of the risk that exists as a function of the *Ls* of the workers under prevention. In the case study (Table 6), the result of 15 indicates that the prior risk estimate or *Cv* for the degree of correction is tolerable. The probability of an accident occurring is medium with low harmful consequences, or the probability of an accident occurring is low with harmful consequences.

### 3.9. Characteristic Values Obtained

**Figure 6:**
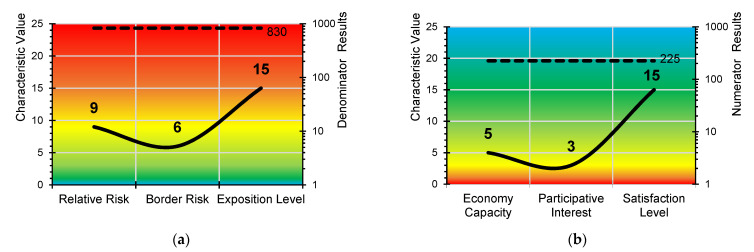
*Cv* Results in the case study: (**a**) Numerator of the *Apac*; (**b**) Denominator of the *Apac*.

The *Cv* of each *Apac* parameter is obtained (Figure 6). Graph (a) shows the results of the numerator of the *Apac,* and Graph (b) shows the values of the denominator. The total obtained in the numerator is 830, and in the denominator, it is 225. Therefore, the *Apac* value will be the quotient between both values, the result of which is 3.69. The degree of correction is not sufficient to correct the situation of constructive complexity. The interpretation of the *Apac* result is based on the absolute risk initially determined in the Occupational Health and Safety Plan for the site. The *Cv* implies that the risk conditions assessed a priori are greater.

## 4. Incidence of the Characteristic Value on the Assessed Risk

Once the *Cvs* have been obtained for each of the *Apac* parameters, the incidence of this *Cv* on the risks to be assessed is analysed. In this study, the most characteristic risks have been selected (Table 7) for building works, following a guideline proportional to the classification established by the Spanish National Institute for Safety and Health at Work [68].

**Figure 7:**
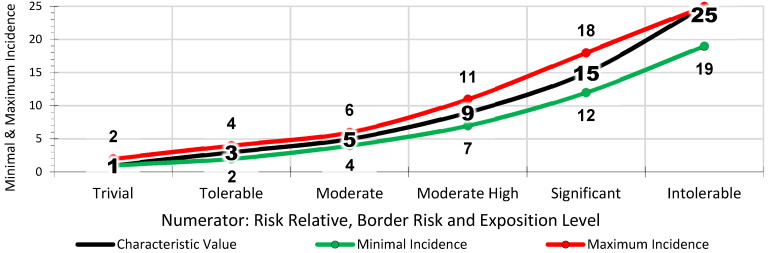
*Cvs* of Incidence of the Numerator Parameters.

**Figure 8:**
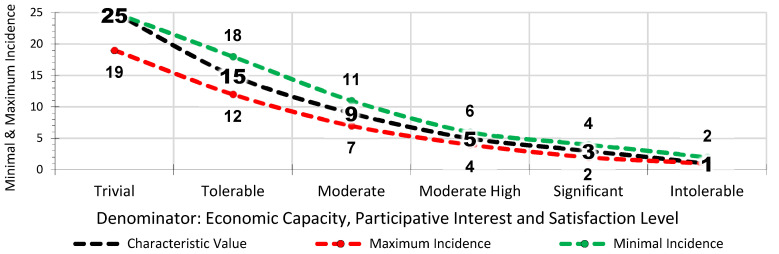
*Cvs* of Incidence of the Denominator Parameters.

Based on the Cv, each of the selected risks to be assessed is analysed to determine whether its incidence is greater or lesser. Thus, in the *Lpac* Methodology, there are two concepts that define the circumstances of the work: the *Cv* of the parameter to be observed, which serves as a basis; and the incidence in the risk to be evaluated based on *Cv*. This implies that the *Cv* can be higher or lower depending on the incidence of the risk to be evaluated (Figure 7), within certain value limits with respect to the incidence. The incidence values are maximum and minimum for each *Cv* (Figure 7 and Figure 8).

This observation is subjective and depends on the technical capacity and experience of the inspection staff. The final results are shown for each of the risks and for each of the parameters of the *Lpac* formula in the case study (Table 8).

## 5. Results

The following graphs show the results of the evaluation of the case study of the unit of work for the construction of an exposed brick façade located on a first floor. This inspection corresponds to the seventh inspection day on 26 June 2016, between 11:00 and 11:37 h.

**Figure 9:**
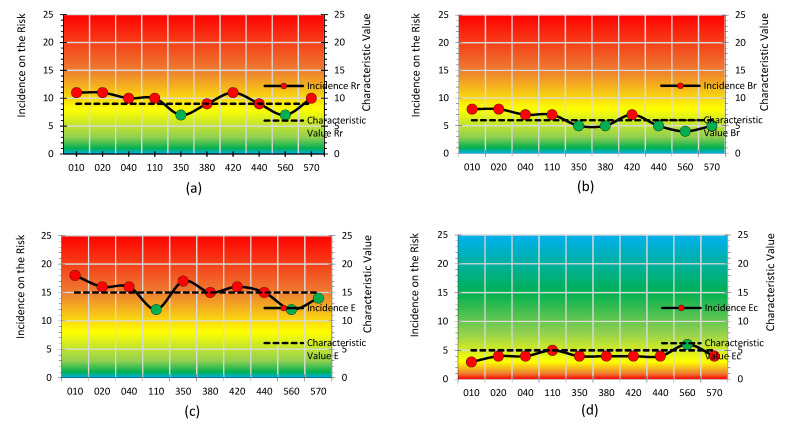

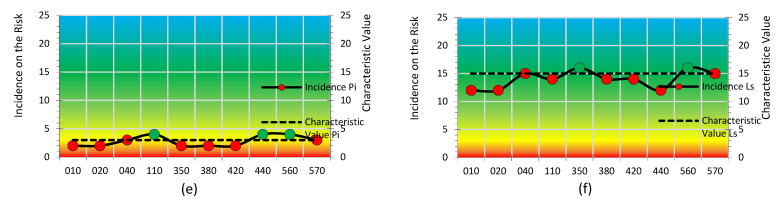
*Cvs* of Incidence of the denominator parameters. (**a**) Risk Assessment for Relative Risk; (**b**) Risk Assessment for Border Risk; (**c**) Risk Assessment for Exposition Level; (**d**) Risk Assessment for Economic Capacity; (**e**) Risk Assessment for Participative Interest; (**f**) Risk Assessment for Level of Satisfaction.

The results are presented vertically, with respect to the *Cv* of each of the *Lpac* parameters and how the incidence of risk varies in each of the assessments made on the defined risks. The graphs show the colour bands identifying the risk estimate for the Cv: trivial (blue), tolerable (green), moderate (light green), moderate high (yellow), notable (orange) and intolerable (red). Figure 9a–c corresponds to the results of the *Apac* parameters of the numerator: *Rr*, *Br* and *E*. Figure 9d–f corresponds to the denominator parameters: *Ec*, *Pi* and *Ls*.

The *Cvs* (dashed line) of the *Rr* and *Br* parameters (Figure 9a,b) fall within the yellow band, and the *E* parameter (Figure 9c) falls within the orange band. However, the parameters of *Ec* and *Pi* (Figure 9d,e) fall within the yellow band and the parameter of *Ls* falls within the green band (Figure 9f). The assessor then makes a subjective estimate of the incidence of risk for each of the risks to be assessed. Considering that there is a higher estimation of risk in Risks 010, 020, 040 (risks of accidents due to people falling to different levels, risks of accidents due to people falling to the same level and risk due to handling loads) and Risk 420 (risk due to displacement) with respect to the ob servation in Safety at Work, and it is lower in Risk 560 (risks due to relations between workers) with respect to the observation in psychosociology.

**Figure 10:**
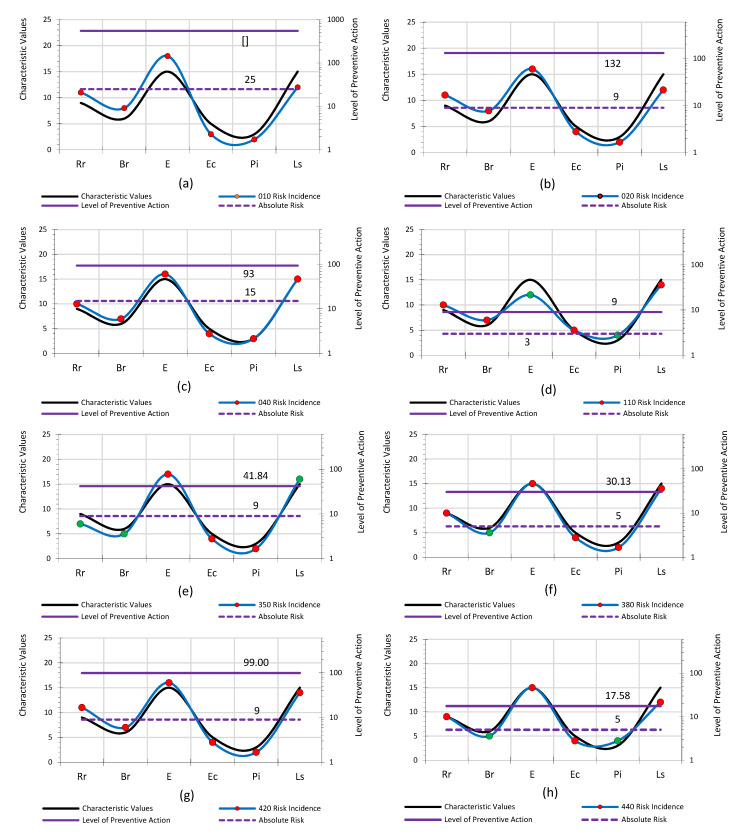

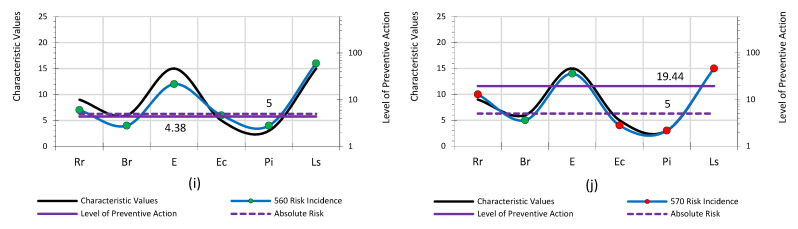
*Cvs* of incidence of the parameters. *Apac*: (**a**) Risk-010, (**b**) Risk-020, (**c**) Risk-040, (**d**) Risk-110, (**e**) Risk-350, (**f**) Risk-380, (**g**) Risk-420, (**h**) Risk-440, (**i**) Risk-560, and (**j**) Risk-570.

Figure 10 presents the results horizontally, showing how the incidence of risk varies with respect to the *Cv* for each of the risks assessed. Each of the graphs shows the result of the characteristic value and its incidence on the risk assessed. Black shows the characteristic value for the first observation and for each of the *Lpac* parameters. Blue shows the impact on the risk in the second observation. The markers indicate whether or not the incidence value increases the risk. The purple dashed line represents the absolute risk value as a basis for comparison. The purple solid line represents the *Lpac* value.

Next, the valuation parameters that correct for the risk environment are those that are placed in the denominator of the *Lpac* formula. This implies that the most restrictive *Cvs* are those with the lowest quantity. In turn, this means that the correction of the given risk levels will be suitable when the quantities are the largest that the *Cv* can take.

Regarding the risk of people falling to different levels (Figure 10a), the risk of people falling to the same level (Figure 10b), the risk of objects falling due to handling (Figure 10c) and the risk due to displacement (Figure 10g), the results show that the impact on the risk assessed is significant. The preventive conditions for each of the parameters in the documentary, constructive and social environments are not adequate (red markers). The parameters of risk exposure, economic capacity, participatory interest and level of satisfaction need to be corrected, as they are real-time parameters and allow for variability. However, the parameters relative risk and frontier risk are fixed parameters that depend on physical and geometrical conditions determined in the documentation.

Concerning the risk of entrapment by or between objects (Figure 10d), the risk of heat stress (Figure 10e), the risk of inadequate lighting (Figure 10f), the risk of incorrect handling of loads (Figure 10h) and the risk of incorrect organisation of work (Figure 10j), the results show that for some parameters the incidence of the assessed risk corrects the *Cv*, which corrects the risk situation (green markers). However, it is necessary to correct the parameters of the constructive (*Ce*) and social (*Se*) environments to achieve a lower *Lpac*.

In relation to the risk due to personal relationships (Figure 10i), the results show that the impact on the risk assessed corrects the *Cv*. With regard to the absolute environment (*Abe*), the value of the *Lpac* is lower, which implies that, compared to the situation foreseen in the risk assessment of the Health and Safety Plan, the real situation analysed is adequate with regard to preventive control.

## 6. Discussion

The results shown in this article relate to the real-time risk assessment of a particular construction site unit. It is important to understand that the characteristic value has a double meaning or is measured in two different approaches. On the one hand, the a priori existing conditions on the construction site are analysed from six different points of view (Apac parameters), obtaining an initial value of the Cv. On the other hand, an analysis is made of how this *Cv* affects the risks that are assessed. Depending on the type of risk, the incidence value may be higher or lower according to the variables shown in the article.

This methodology may seem complex due to the large number of parameters that the mathematical formula has. However, all the parameters defined in the method correspond to the actual routine of the technician when he performs a construction site inspection. The documentation is always previously analyzed to study the construction systems and the way to build effectively (in time, form, and economy). They always go to the construction site to see how the construction systems are proceeding and how the workers have high performance in their work development. The fundamental approach of this methodology is to propose a change of observation and introduce the concept of preventive environment as one more element in the routine of the technician (in this case of the prevention technician). From this point of view, the documentation will be analyzed previously, focusing on the health and safety circumstances (or the documentary environment) that accompany each of the construction systems. When the work is visited, the health and safety circumstances (or construction environment) that accompany the way in which the project is being implemented, how the construction systems are being developed, how the preventive systems are being developed and for how long, should be analyzed. Workers are exposed to risk while the project is being implemented. Another element, which is not usually considered, is the observation of the health and safety circumstances (or social environment) in the face of personal relationships and role interaction; it is essential to check that all workers (white collar and blue collar) understand the information and health and safety training they receive. 

One of the fundamental preventive environments of this method is the verification and analysis of the social environment, with the parameters of participatory interest (*Pi*) and level of satisfaction (*Ls*). Both parameters are conditioning factors in the correction factor of the formula. The absolute environment (*P* and *C*) is defined in the Health and Safety Plan prior to construction; the documentary environment (*Rr* and *Br*) which is defined by the qualification of the worker, the physical conditions of the material and the geometry of the building; and the construction environment (*E* and *Ec*) that depends on the working conditions imposed by the employer and the contracted economic conditions. They are conditions imposed and with little variation during the implementation of the project. However, the social environment (*Pi* and *Ls*), which depends on the social conditions of the individual and the group, establishes, in a decisive way, that the result of the Level of Preventive Action (*Lpac*) is favorable or unfavorable. Therefore, it is essential to verify that workers actively participate in prevention, not only individually, but collectively. This is with which one of the fundamental concepts of risk prevention in construction works is applied: putting collective protection before individual protection. Workers must understand that informing about risk prevention training is decisive. The evaluator must consider that good data collection implies establishing communication with the workers. From these communications, it can be concluded about the participatory interest that workers have in risk prevention.

The procedure or protocol of this new workplace risk assessment methodology adapted to construction sites can be implemented on a computer application that facilitates data collection and provides the results graphically. Currently, this methodology is being applied in various construction sites (in Spain, Portugal, and Brazil). With the results obtained, the bases for the development of a computer support will be determined. This is to ensure that the risk assessment is carried out at the construction site in real time and with immediate results. Currently, too, research on human behavior and the prediction of its movements are being implemented on the new methodology. In this case, about workers on a construction site. New communication technologies can facilitate the difficult task of the preventionist.

In this way, the risks must be seen not as fixed contexts, but as variable contexts that depend on very different conditions. This variability of results can explain why risks are seen and analysed in very different ways by evaluators. Depending on the point of view or the observed preventive environment (*De*, *Ce* and *Se*), the danger may be more intense or more evident in certain assessment risks. Therefore, in this situation, it is important to learn to identify and observe the utopian context of non-risk versus the obvious context of hazard [69].

## 7. Conclusions

The risk assessment method of the Level of Preventive Action integrates the different environments of the development of a building, the documentary, constructive and social environment; offering an assessment in the constructive phases with the work project, the management of the work and the safety climate of the work, covering the possibility of assessing the corresponding risks.

It also includes a new assessment concept called “Characteristic Value”, associated with the real characteristics of the work unit under execution, which is based on the criteria of complexity of the work unit, the position of the work unit, exposure to risk, organisational procedure, prevention participation and congruence of the perception of risk. This determines an a priori minimum value for the risk associated with the actual conditions of the site unit.

With the incidence of the Characteristic Value in the risk of observation, the evaluation is specialised, obtaining results that are more in line with the reality that is appreciated and with a range of values that cover a multitude of possibilities; which can be adapted to any of the situations that occur during the building process.

The method is protocolised on the basis of technical-social criteria whose parameters are close to any construction site situation in the fields of Occupational Safety, Industrial Hygiene, Ergonomics and Psychosociology. In each of the phases of the methodology protocol, a very precise evaluation is carried out. Each value obtained in one phase determines quantitatively the value in the next phase and, qualitatively, the result of the final preventive action control. This procedure ensures that the value and preventive control obtained are easily interpreted by the evaluator. In addition, a result is achieved that is adapted to the real circumstances of the construction. This value or result is achieved quickly once the inspection has been carried out; unlike other particular methods that require further work for the interpretation of the result. It is convenient to remember the changing circumstances and particular conditions of the construction methods and systems during the project implementation stage. Finally, the result of the preventive action control can be transferred to the real and current needs of the work, immediately. This means that it is a very flexible method in its applicability and more sensitive to detect risks in all situations in the construction process.

It is true that the leader of a company, the boss, the manager, and the teams that occupy the high-ranking positions are decisive part in terms of risk prevention. This new methodology performs an analysis in the absolute environment that is defined by the initial documentation (Occupational Health and Safety Plan). However, Characteristic Values of the documentary environment with the relative risk and border risk are a consequence of the initial design: Regarding decision-making in the conception of the building: design, budget, construction, development of the work. For this reason, this analysis involves the evaluation of the documentation defined by the project team. Of course, the degree of exposure to risk and the economic capacity in the construction environment depend on the decisions of the management team. Further, because of the leadership regarding the essential training and information on risk prevention, the method assesses the participation in prevention and the workers’ emotional state. The true attitude of leadership regarding safety is decisive in an organization.

Finally, the method aims to increase the awareness of workers and site agents in prevention systems by means of a practical procedure, which allows economic savings both for development companies, builders and the self-employed, as well as for the health sector (Social Safety and Private Health).

## Figures and Tables

**Table 1:** Level of Preventive Action. Theoretical–Mathematical Foundations.

Construction Stages				
1st	2nd	3rd	4th	5th
**Associated Risk Phases**				
Initial Design	Project Drafting	Contractor Contract	Project Implementation	Use and Maintenance
**Construction Risk Analysis Parameters**				
Traditional Risk Analysis	Physical and Geometrical	Construction Resources	Emotional States	Use and Maintenance
**Building Preventive Environment**				
Absolute *Abe*	Documentary *De*	Construction *Ce*	Social *Se*	Life Cycle *Lce*
**Construction Preventive Environment**				
*Lpac = (Abe) · (De) · (Ce) · (Se)*				
**Level of Preventive Action Parameters**				
Probability *P*	Relative Risk *Rr*	Exposure Degree *E*	Participative Interest *Pi*	
Consequences *C*	Risk of Border *Br*	Economic Capacity *Ec*	Level of Satisfaction *Ls*	
**Absolute Risk**	**Assessment of Preventive Action Parameters**			
*Abr =* *(P·C)*	*Apac = ((Rr · Br · E)/(Ec · Pi · Ls))*			
Level of Preventive Action Formula				
*Lpac = (P · C) · ((Rr · Br · E)/(Ec · Pi · Ls)) = Abr · Apac*				

The Meaning of the Abbreviations Can Be Seen in Appendix A (Table A1).

**Table 2:** Methodology Level of Preventive Action. Scheme of the Protocol.

	First Phase. Characteristic Value *(Cv)*											
1st	**Construction Preventive Environment**											
	**Absolute *(Abe)***			**Documentary *(De)***			**Construction *(Ce)***			**Social *(Se)***		
	Defined in the Project			Specialized Technical Assessment						Survey in the Workplace		
	*P*	*C*		*Rr*		*Br*	*E*	*Ec*		*Pi*		*Ls*
	1-3-5-9-15-25	1-3-5-9-15-25		1-3-5-9-15-25		1-3-5-9-15-25	1-3-5-9-15-25	25-15-9-5-3-1		25-15-9-5-3-1		25-15-9-5-3-1
	**Second Phase. Incidence on the Assessed Risk**											
2nd	Work Safety Risks			Industrial Hygiene Risks			Ergonomic Risks			Psychosociology Risks		
	*Rr-Br-E-Ec-Pi-Ls*			*Rr-Br-E-Ec-Pi-Ls*			*Rr-Br-E-Ec-Pi-Ls*			*Rr-Br-E-Ec-Pi-Ls*		
	**Third Phase. Basis of Prevention Control**											
3rd	*Lpac (%) = (Abr) · (Apac)*											
	Lpac < 4%		4% < Lpac < 12%		12% < Lpac < 24%		24% < Lpac < 36%		36% < Lpac < 60%		Lpac > 60%	
	Optimal Control		Adequate Control		More Control		Greater Control		Intensive Control		Exhaustive Control	
	**Fourth Phase. Recommendation Actions**											
4th	Amount of Preventive Action Control Required											
	Risks Assessment Work Safety Risks Industrial Hygiene Risks Ergonomic Risks Psychosociology Risks				Environmental Assessment Absolute Documentary Constructive Social				Workers Assessment One Worker Worker Teams Technicians Global Work			
	**Fifth Phase. Checking the Improvement of Preventive Actions**											
5th	Amount of Preventive Action Control Implemented											
	Risks Assessment				Environmental Assessment				Workers Assessment			

The Meaning of the Abbreviations Can Be Seen in Appendix A (Table A1).

**Table 3:** Characteristic Values: Relative Risk, Border Risk and Exposure Risk.

**Risk Estimation** **Relative Risk, Border Risk and Exposure Risk**			**Severity of the Consequences**					
			**Slightly Damaging**		**Damaging**		**Extremely Damaging**	
			**1**		**3**		**5**	
**Probability**	**Low**	**1**	Trivial	1	Tolerable	3	Moderate low	5
	**Medium**	**3**	Tolerable	3	Moderate high	9	Significant	15
	**High**	**5**	Moderate low	5	Significant	15	Intolerable	25

**Table 4:** Characteristic values: economic capacity, participative interest and satisfaction level.

**Risk Estimation** **Economic Capacity, Participative Interest and Satisfaction Level**			**Severity of the Consequences**					
			**Slightly Damaging**		**Damaging**		**Extremely Damaging**	
			**5**		**3**		**1**	
**Probability**	**Low**	**5**	Trivial	25	Tolerable	15	Moderate low	5
	**Medium**	**3**	Tolerable	15	Moderate high	9	Significant	3
	**High**	**1**	Moderate low	5	Significant	3	Intolerable	1

**Table 5:** Interpretation of the health and safety plan assessment.

Risk to Assess		Health and Safety Plan. Assessment Company	Assessor’s Assessment		
			Consequence	Probability	Absolute Risk
Code	Description		*C*	*P*	*Abr*
010	Risk of people falling from a different height	Intolerable	5	5	25
020	Risk of people falling from the same height	Moderate	3	3	9
040	Risk of objects falling during handling	Significant	3	5	15
110	Risk of entrapment by or between objects	Tolerable	1	3	3
350	Risk due to thermal stress	Moderate	3	3	9
380	Risk due to inadequate lighting	Moderate	1	5	5
420	Risk due to movement	Moderate	3	3	9
440	Risk due to incorrect load handling	Moderate	1	5	5
560	Risk due to personal relationships	Moderate	1	5	5
570	Risk due to incorrect work organization	Tolerable	1	3	3

The Meaning of the Abbreviations Can Be Seen in Appendix A (Table A1).

**Table 6:** *Lpac* assessment for the case study.

**Apac**	**Observational Analysis**								**Mean**	**Cv**
**Relative** **Risk**	Graphism	Setting-out	Workers	Qualification	Work Plan	Machines	Weight	Manageable		
	15	5	9	5	15	5	3	1	7.3	9
**Border Risk**	Height	Border								
	<5 m	<100 cm								
	6	6							6	6
**Degree** **of** **Exposure**	Intensity	Repetition								
	Internal Risk, External Risk	Exposure > 5 Remarks								
	15	9							12	15
**Economic** **Capacity**	Individual Organisation	Group Organisation	Global Work Organisation	Equipment Personal Protection	Equipment Collective Protection					
	3	9	5	5	1				4.6	5
**Participatory** **Interest**	Information	Individual Participation	Group Participation	External Appearance						
	5	3	3	1					3.5	3
**Level of** **Satisfaction**	State of mind	Safety Perception	Perception of the Environment							
	13	9	15						13	15

**Table 7:** Selected characteristic risks.

Discipline	Code	Risk to Assess
Safety at Work	010	Risk of people falling to another level
	020	Risk of people falling on the same level
	040	Risk of falling objects due to handling
	110	Risk of entrapment by or between objects
Industrial Hygiene	350	Risk of heat stress
	380	Risk due to inadequate lighting
Ergonomics	420	Displacement risk
	440	Risk due to incorrect handling of loads
Psychosociology	560	Risk due to personal relationships
	570	Risk of incorrect organisation of work

**Table 8:** Incidence of *Cv* on risk.

Risks to Assess		Preventive Environmental							Assessment of Preventive Action *Apac*	Level of Preventive Action *Lpac (%)*
		Absolute *Abe*	Documentary *De*		Constructive *Ce*		Social *Se*			
		*P·C*	Characteristic Value (*Cv*)							
			*Rr*	*Br*	*E*	*Ec*	*Pi*	*Ls*		
			9	6	15	5	3	15		
Code	Risk Description		Incidence on Risk							
010	Different Level	25	11	8	18	3	2	12	22	88%
020	Same Level	9	11	8	16	4	2	12	15	163%
040	Handling of Loads	15	10	7	16	4	3	15	6	25%
110	Entrapment	3	10	7	12	5	4	14	3	100%
350	Thermal	9	7	5	17	4	2	16	5	52%
380	Lighting	5	9	5	15	4	2	14	6	121%
420	Displacement	9	11	7	16	4	2	14	11	122%
440	Loads	5	9	5	15	4	4	12	4	70%
560	Relationships	5	7	4	12	6	4	16	1	18%
570	Organisation	5	10	5	14	4	3	15	4	78%

The Meaning of the Abbreviations Can Be Seen in Appendix A (Table A1).

## Data Availability

The data presented in this study are available upon request from the corresponding author. The data are not yet publicly available because the authors are still using them for research purposes.

## Appendix A

Table 1 and Table 2 (Section 2.1. Theoretical—Mathematical Foundations) show the different parameters that are used in this occupational risk assessment methodology. However, below is a list of the acronyms used in this article:

**Table A1:** List of acronyms used in the article.

**Acronym**	**Description**
*Lpac*	Level of Preventive Action
*Apac*	Assessment of Preventive Action
*Cv*	Characteristic Value
*Cvs*	Characteristic Values
*Abe*	Absolute Environment
*De*	Documentary Environment
*Ce*	Constructive Environment
*Se*	Social Environment
*Lce*	Life Cycle Environment
*P*	Probability
*C*	Consequence
*Abr*	Absolute Risk
*Rr*	Relative Risk
*Br*	Border Risk
*E*	Exposure Level
*Ec*	Economic Capacity
*Pi*	Participative Interest
*Ls*	Level of Satisfaction
*INSST*	National Institute of Safety and Health at Work in Spain
